# Comparing Preferred Temperatures and Evaporative Water Loss Rates in Two Syntopic Populations of Lacertid Lizard Species

**DOI:** 10.3390/ani14243642

**Published:** 2024-12-17

**Authors:** Jelena Ćorović, Nada Ćosić, Jelka Crnobrnja-Isailović

**Affiliations:** 1 Department of Evolutionary Biology, Institute for Biological Research “Siniša Stanković”—National Institute of the Republic of Serbia, University of Belgrade, Despota Stefana Boulevard 142, 11000 Belgrade, Serbia; nada.cosic@ibiss.bg.ac.rs (N.Ć.); jelka@ibiss.bg.ac.rs (J.C.-I.); 2 Department of Biology and Ecology, Faculty of Sciences and Mathematics, University of Niš, Višegradska 33, 18000 Niš, Serbia

**Keywords:** thermoregulation, preferred temperatures, evaporative water loss, peripheral populations, Lacertidae, *Darevskia praticola*, *Podarcis muralis*

## Abstract

Many reptiles actively regulate their body temperature. During thermoregulation, they suffer evaporative water loss (EWL). Since evaporation increases with temperature, EWL could limit the activity of ectotherms when water is not available. In this study, we compared the preferred body temperatures (T_p_) and EWL of two lacertid lizard species, *Darevskia praticola* and *Podarcis muralis*, at the western edge of *D. praticola*’s range, where they live in syntopy. We hypothesized that *D. praticola*, a species that inhabits forested and humid environments, would have a higher EWL than the more widespread *P. muralis*. Our results show that *D. praticola* prefers lower temperatures (mean T_p_ = 28.1 °C) than *P. muralis* (mean T_p_ = 30.6 °C). Despite the differences in their thermal preferences, both species showed similar total EWL (2.76% for *D. praticola* and 2.67% for *P. muralis*), although their daily patterns of water loss differed. Our results suggest that *D. praticola* has developed mechanisms to control water loss and that its lower thermal preference may be due to both historical factors and local adaptations. These results contribute to the understanding of how environmental factors influence the physiology of lizards, which in turn has implications for predicting the effects of climate change on species distribution.

## Simple Summary

Many reptiles actively regulate their body temperature and during thermoregulation, they lose water from the body. The amount of body water lost in maintaining an adequate body temperature can vary from species to species. In this study, we compared the preferred body temperatures and the amount of water loss in males of two lacertid lizard species—the meadow lizard (*Darevskia praticola*) and the common wall lizard (*Podarcis muralis*)—that share the habitat at the western edge of the meadow lizard’s distribution area. We hypothesized that the meadow lizard would exhibit higher water loss than the common wall lizard, as it generally prefers humid and forested places. However, the results showed that, at this locality, water loss is similar for both species, although the meadow lizard preferred lower ambient temperatures than the common wall lizard. We concluded that the meadow lizard has developed mechanisms to control water loss. Its preference for lower temperatures could be due to both historical factors and local adaptations. This information could help us to better understand how lizards cope with environmental changes and, more importantly, what we should do to prevent the species’ decline in the wake of climate change.

## 1. Introduction

Reptiles rely on external sources of heat to achieve body temperatures necessary for their physiological processes, as well as other activities such as feeding, avoiding predators, and reproducing [1,2,3]. Additionally, numerous studies have shown that habitat humidity is another important environmental factor for terrestrial reptiles and that the interaction of ambient humidity and temperature has been shaping activity patterns and the distribution of many species [4,5,6,7]. Many reptiles have the ability to actively regulate their body temperature in response to varying environmental conditions. They achieve this by altering their behavior, adjusting their activity patterns, and selecting appropriate microhabitats [8,9,10]. However, during thermoregulation, they suffer evaporative water loss (EWL) mainly through the skin but also through respiratory passages and the eyes [11,12]. Since evaporation increases with temperature, a trade-off between thermoregulation and water balance may exist where EWL could constrain the activity of ectotherms when water is not available [6,13,14,15]. Mechanisms that limit the water loss from the body are essential for survival in terrestrial environments, and the intensity of water loss greatly depends on the temperature and the humidity of the habitat [11,12].

Preferred body temperature (T_p_) and evaporative water loss (EWL) are two ecophysiological parameters that have often been used to describe lizards’ responses to the environment. The first one represents the range of temperatures selected by lizards in the absence of thermoregulatory constraints [16]. This temperature range is an important indicator of the thermal preference of the species because it is correlated with the optimal performance of many physiological functions [3,17,18]. Thermal preference of a species can remain similar under different environmental conditions and over longer time scales [3,19,20,21], but it has also been observed that a variety of factors, such as seasonality (especially in temperate species of lizards), ontogeny, or reproductive status, can influence the preferred body temperatures [8,18,20,22]. The evaporative water loss (EWL) provides evidence of the degree of resistance to water loss. According to Mautz [11], EWL (in the absence of defecation) and metabolic gas exchange (which is usually negligible) are reflected in the body weight loss; therefore, following the body weight loss is the most common method used for measuring the water loss rate (for a recent review of methods used in squamate reptiles, see Le Galliard et al. [23]). These ecophysiological measurements can be used to assess the thermal preference of individuals and their capacity to resist water loss, but they can also determine if these traits differ between populations or species [5,24,25,26,27].

Rapid global climate change poses significant extinction risks for reptiles [28,29,30]. If effective mitigation measures are not implemented, reptiles may experience large-scale declines in the near future due to climate change [30]. Understanding the relationship between species’ thermal requirements and geographic gradients is crucial for predicting their vulnerability to extinction from climate change [31]. Some niche-specialist lizard species may be able to adapt to changing environmental conditions successfully, provided they possess sufficient physiological plasticity [32]. A predicted rise in temperature and decrease in rainfall in southern and central parts of Europe will affect the availability of water for organisms [33,34]. In addition, climate warming will also increase the evaporation rates of organisms [6,29]. Hence, an integrated approach that takes into account these ecophysiology data can be highly useful in developing mechanistic distribution models. These models can be used to predict the distribution and the degree of vulnerability of species under climate change and provide directions for future conservation measures [6,35,36,37,38]. It is important to note that these changes can have different impacts at the edges of a species’ range, where populations tend to be smaller and more fragmented. As species approach their ecological limits, this fragmentation may lead to genetic isolation, reducing the potential for local adaptation [39,40].

In this study, we examined T_p_ and EWL in syntopic populations of two lacertid species, *Darevskia praticola* and *Podarcis muralis*, with an emphasis on *D. praticola*. There is a general scarcity of data on thermal physiology and water loss regarding the whole *Darevskia* genus. Until now, only three studies have been published on the thermal ecology of 4 *Darevskia* species—*D. praticola* in Serbia [41], *D. valentini* in Armenia [42], *D. armeniaca*, *D. unisexualis,* and *D. valentini* in Armenia [43], plus an extensive study on climate variation shaping diversification and genome variation in lacertid lizards [7] where a few basic thermal and water loss data of *D. praticola*, *D. rudis,* and *D. valentini* were included. This study was conducted on *D. praticola* because there is little data on its ecophysiology, and therefore it should be studied in more detail. Since *P. muralis* is the only small lacertid found in syntopy with *D. praticola* at the western edge of the distribution area, we have used it as a reference point for comparison. *Podarcis muralis* has previously been an object of studies concerning the thermal preferences of the species [17,44,45,46,47,48] and combined studies of thermal ecology and water loss [24,49].

Our goal was to investigate certain ecophysiological characteristics of a species at the edge of its distribution range living in syntopy with a potential competitor. Our main hypothesis was that *D. praticola* has higher water loss rates than the widespread *P. muralis,* considering its preference toward forested and humid habitats. Additionally, we supposed that *D. praticola* does not show significant interannual variation in preferred temperatures. Therefore, we experimentally analyzed T_p_ and EWL in both species to (1) evaluate the differences between the species in T_p_ and variation of T_p_; (2) examine whether there is a difference in T_p_ of *D. praticola*, compared to our previous research at the same study site; (3) determine EWL rates and their daily variation in both species.

## 2. Materials and Methods

### 2.1. Analyzed Species

**Figure 1:**
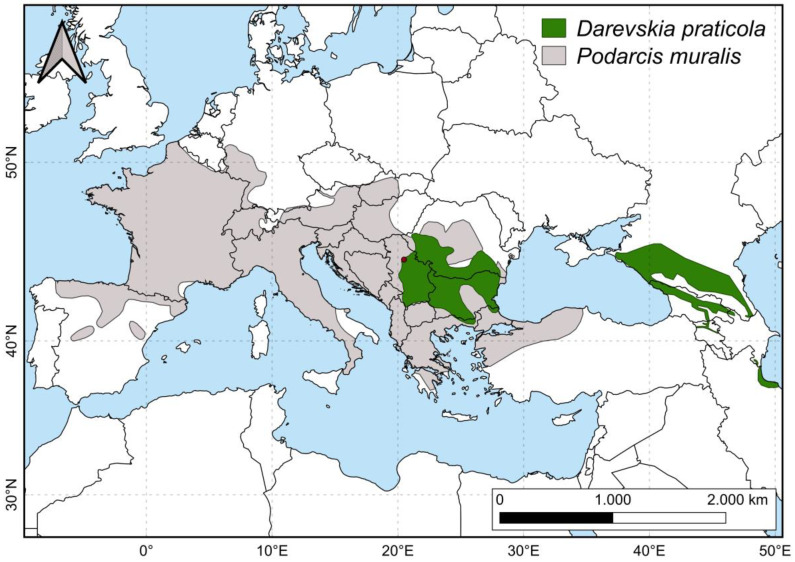
Distribution ranges of *Darevskia praticola* and *Podarcis muralis*. The red dot on the map represents the geographic position of this study locality. The shapefiles used for the map were downloaded from the IUCN Red List of Threatened Species [50,52].

Both analyzed species are small lacertid lizards with partially overlapping ranges in the Balkans (Figure 1): *P. muralis* is distributed from the Iberian Peninsula to Asia Minor and is widespread across Central and Southern Europe [50,51], while *D. praticola* has a disjunct distribution, with its range split into two subranges—Eastern (Caucasus) and Western (Eastern and South-Eastern Europe) [51,52]. The western portion of its range is in Southeastern Europe (covering areas in Romania, Serbia, Bulgaria, Northern Greece, and the European part of Turkey), where the species reaches its western distribution limit in Central Serbia (Ćorović et al. [53] and references therein). *Podarcis muralis* is widespread through most of its range and can be found in a wide variety of habitats, from sunny slopes in broad-leaved and coniferous forests to fields, rocky surfaces, and stone walls, and also in urban environments [54,55]. It can sometimes be found syntopic with *D. praticola*. Unlike other species in its genus, which are typically saxicolous, *D. praticola* is a ground-dwelling lizard. It is most commonly found in open oak woodlands with a well-developed herbaceous understory and tends to avoid dry forest habitats [56,57,58,59]. In cases of deforestation and habitat degradation, it can be found in the humid, grassy vegetation surrounding streams or drainage canals [60]. Overall, *D. praticola* prefers moister and more shaded environments compared to other small lacertids [56,60,61,62]. More detailed information on the distribution and habitat preference of *D. praticola* can be found in the ecological niche study of Ćorović et al. [53].

### 2.2. Field Procedures

Ten adult males of both species (*D. praticola* and *P. muralis*) were captured by noose during the reproductive season in May of 2019 at the same study site—the slopes of Avala mountain, near Belgrade, Central Serbia (44°40′52.5″ N, 20°33′00.3″ E; 230 m altitude), overgrown with a forest of a thermophilic oak community *Quercetum frainetto-cerris*. This study locality is set at the westernmost edge of *D. praticola’s* range, characterized by higher mean annual temperatures and lower amounts of annual precipitation compared to central habitats of *D. praticola* in this area (eastern Serbia and southwestern Romania), and it is under intense habitat fragmentation caused by human activity [41].

### 2.3. Preferred Body Temperatures

In this experiment, we followed the procedure described in Carretero, Roig, and Llorente [20] and Veríssimo and Carretero [63], following the same approach as in our previous study on the thermal ecology of *D. praticola* [41]. To eliminate the potential effects of reproduction, body condition, and ontogeny on T_p_ and EWL, we only analyzed adult males (*D. praticola*: N = 10 and *P. muralis*: N = 10) [20,27]. The lizards were housed in individual cages under natural light conditions for no more than three days prior to the experiment, with *ad libitum* food and water. Snout-vent length (SVL) of every lizard was measured to the nearest 0.01 mm using callipers, and weight was measured to the nearest 0.001 g using an analytical scale. Only lizards with unbroken or fully regenerated tails were included. Each lizard was individually exposed to a thermal gradient (~20–45 °C) created by a 150 W infrared reflector bulb positioned at one end of the terrarium (100 × 40 × 30 cm) [60]. The terraria were placed in a room with a natural light photoperiod and a stable temperature of around 20 °C. The bulbs were turned on at 07:00 h, and the lizards were placed in the experimental terraria at 08:00 h, with the first measurements taken at 09:00 h [20]. Preferred body temperature (T_p_) was measured hourly from 09:00 to 17:00 h by inserting the tip of an electronic digital thermometer into the cloaca (Dostmann digital Einstich–Thermometer TFA with an accuracy of ±0.1 °C) within 10 s of catching each individual to minimize heat transfer from the researcher’s hand [63]. The thermal gradient was monitored hourly throughout the experiment. Set-point temperature ranges (T_set_) were estimated for each lizard as the central 50% of all body temperatures (T_b_) selected in the thermogradient [16].

### 2.4. Evaporative Water Loss Rates

The evaporative water loss experiments were conducted the day after the thermal experiments (following Osojnik et al. [24]; Carneiro et al. [49]; and Carneiro et al. [5]). During the thermal experiments, the lizards were not fed, and after the experiment, until the next day, they only had access to water. This way the lizards could rehydrate, but the possibility of defecation that could affect the results of the water loss experiment was diminished. After the experiments, the lizards had a recuperation period with food and water, after which they were released at their capture sites.

During the water loss experiment, individual lizards were placed in opaque plastic boxes (12 cm × 7 cm × 8 cm) with a perforated lid and bottom to enable air circulation. Under each individual box was an additional box, containing silica gel (5 g) that maintained a lower relative humidity in the box with the lizard. Groups of four individual boxes were placed in a bigger (40 cm × 30 cm × 19 cm), closed box. The bigger box also contained a solid calcium chloride moisture absorber that was used to keep the relative humidity in the box between 20 and 30%. The temperature of the experimental room was set to 24 °C because it falls around the low threshold of activity of most lacertids [18], so the activity of animals and the amount of stress were reduced, but the evaporative water loss was still enabled. Relative humidity and temperature were monitored in the box to the nearest 0.1% and 0.1 °C, respectively, using a Testo 410-2 hygrothermometer (Testo, Neustadt, Germany). The experiment was conducted from 09:00 to 17:00 h for eight consecutive hours (covering the daily activity period of the species, based on previous field experience at the chosen locality). To avoid stress from handling, the lizards were weighed in their individual boxes, at hourly intervals, with an analytical scale with ±0.001 g precision. Each weighing operation took around 10 s to minimize the disturbance of animals [5]. The lizard’s body mass during the experiment was calculated by subtracting the weight of the corresponding box that was individually marked and measured before the experiment, to the precision of ±0.001 g, from the weight of the box with the lizard. Before the analyses, the data were checked for errors and outliers.

Using these data, two measures of the relative water loss were calculated. Accumulated evaporative water loss—EWL_a_, which enables the assessment of the total water loss (EWL_t_) during the experiment and is calculated for every hour of the experiment (from 10:00 to 17:00 h) using the formula: EWL_a_ = [(W_0_ − W_n_)/W_0_] × 100. The second measure is the instantaneous evaporative water loss—EWL_i_ which shows the water loss between two consecutive measurements, and it can be used to see if the pattern of water loss changes during the experiment. Instantaneous evaporative water loss is calculated using the formula: EWL_i_ = [(W_n_ − W_n+1_)/W_0_] × 100. In both formulas, the W_0_ is the initial mass before the experiment (at 09:00 h), W_n_ is the mass at a certain time of measurement, and the acquired values are represented in percentages.

### 2.5. Statistical Analyses

We first used GLM (co)variance analyses with repeated measurements to determine the variation in T_p_ according to species and time interval (within-subject factor). In the second step, SVL and body mass of the lizards were incorporated as covariates to account for the effect of size and shape. For water loss experiments, we also used these analyses to determine the differences in instantaneous water loss (EWL_i_) between species and time intervals, adding as covariates the SVL and body masses of lizards. We also calculated the accumulated water loss for the eight intervals (EWL_a_) and compared it between species using GLM (co)variance analyses, with SVL and body mass as covariates. The possible trade-off between T_p_ and EWL was investigated through Spearman’s correlation between mean T_p_ and total EWL ([(W_0_ − W_8_)/W_0_] × 100) separately for both species. We also assessed the influence of SVL and body mass on mean T_p_ and the total EWL. All the analyses were performed in Statistica 12 software [64].

## 3. Results

### 3.1. Species Morphology

The SVL of adult males used in this experiment was statistically different between the two species (*D. praticola*: 48.65 ± 1.70 mm, *P. muralis*: 61.30 ± 2.85 mm; univariate tests of significance, F_1,18_ = 145.45; *p* < 0.001) and body mass (W_0_) (*D. praticola*: 2.35 ± 0.24 g, *P. muralis*: 4.95 ± 0.57 g; univariate test of significance, F_1,18_ = 175.23, *p* < 0.001) (Table 1). Males of *P. muralis* were larger and heavier than males of *D. praticola*.

### 3.2. Preferred Body Temperature

**Figure 2:**
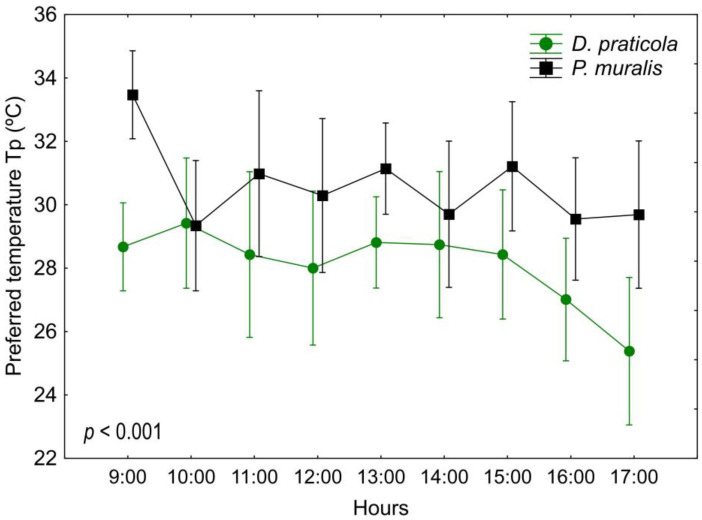
Daily variation of the preferred body temperatures (T_p_) of *Darevskia praticola* and *Podarcis muralis*, showing a different pattern between the two species. Displayed are the mean values and 0.95 confidence intervals. The presented *p*-value illustrates the magnitude of the difference in T_p_ between the species.

Results of preferred body temperatures (Table 1 and Table A1, Figure 2) showed a significant difference between the two species (F_1,18_ = 18.98; *p* < 0.001) and a significant variation among time intervals with a trend of decrease of T_p_ in *D. praticola* (F_8,144_ = 2.19; *p* < 0.05). The interaction of time*species showed no difference (F_8,144_ = 1.19; *p* = 0.311). Incorporating SVL and W_0_ as covariates had an influence on these results (species F_1,16_ = 1.13; *p* = 0.305; time F_8,128_ = 0.51; *p* = 0.846; time*species F_8,128_ = 1.28; *p* = 0.261). There were no significant correlations between SVL and W_0_ with the mean T_p_ in either of the species.

Set-point range (T_set_) calculated as the interquartile 50% from all preferred temperatures varied between 26.9 °C and 29.4 °C, with a mean of 28.1 ± 2.5 °C for *D. praticola*, and between 29.0 °C and 32.7 °C, with a mean of 30.6 ± 3.8 °C for *P. muralis*. To gain a better insight into the thermal preferences of *D. praticola*, we have also taken into account data from our earlier experiments—from 2014 [38] and unpublished data from 2018 [62] (Table 2). Ambient temperatures, corresponding to the month of the experiments, were—June 2014 (min–max: 16.4–26.4 °C, mean: 21.4 °C), May 2018 (min–max: 16.2–26.8 °C, mean: 21.5 °C), and May 2019 (min–max: 11.6–20.2 °C, mean: 15.6 °C) (data obtained from a local weather station).

### 3.3. Evaporative Water Loss

**Figure 3:**
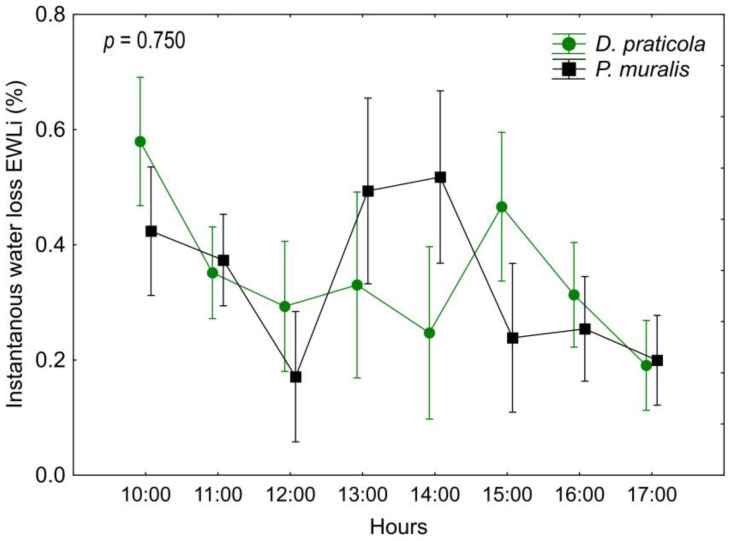
Daily variation of the instantaneous water loss (EWL_i_) of *Darevskia praticola* and *Podarcis muralis*, showing a different pattern between the two species. Displayed are the mean values and 0.95 confidence intervals. The presented *p*-value illustrates the magnitude of the difference in EWL_i_ between the species.

Our analysis of EWL_i_ rate (Table A2, Figure 3) showed a difference between time intervals and in the interaction of time*species (time F_7,126_ = 7.26; *p* < 0.001; time*species F_7,126_ = 5.05; *p* < 0.001) but not between the species (F_1,18_ = 0.10; *p* = 0.750), indicating that the species have different EWL patterns throughout the day.

**Figure 4:**
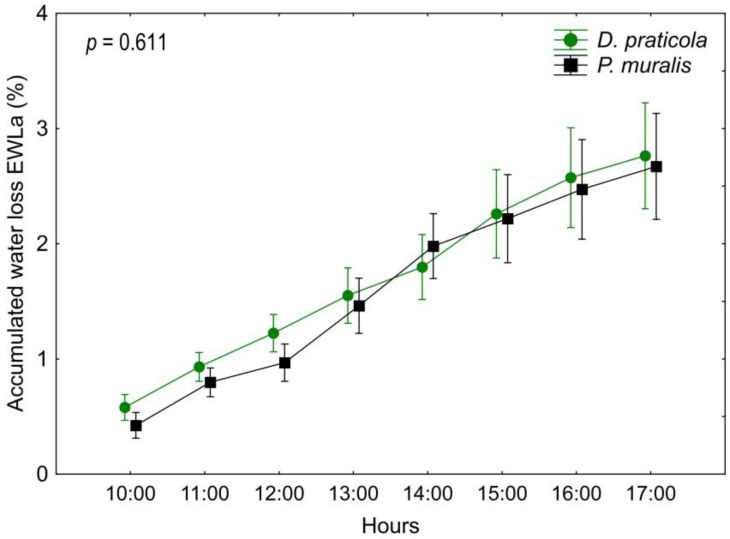
Daily variation of the accumulated water loss (EWL_a_) of *Darevskia praticola* and *Podarcis muralis* shows a similar pattern for the two species. Displayed are the mean values and 0.95 confidence intervals. The presented *p*-value illustrates the magnitude of the difference in EWL_a_ between the species.

Rate of EWL_a_ (Table A3, Figure 4) as a function of species and interaction of time*species did not show a significant difference (species F_1,18_ = 0.27; *p* = 0.611; time*species F_7,126_ = 0.98; *p* = 0.452), but as EWL_a_ increases over time, the influence of time was significant (F_7,126_ = 161.98; *p* < 0.001). Adding SVL and W_0_ as covariates had no influence on the results of the EWL_a_ analyses (species F_1,16_ = 1.78; *p* = 0.201; time F_7,112_ = 4.762; *p* < 0.001; time*species F_7,112_ = 0.71; *p* = 0.663).

Analysis of EWL_t_ rate did not show a significant difference between the species (F_1,18_ = 0.089; *p* = 0.770). Moreover, there were no significant correlations between SVL and the EWL_t_ in either of the species. Total EWL was correlated with W_0_ only in *D. praticola* (R = −0.7; *p* < 0.05). Total EWL for *D. praticola* was 2.76 ± 0.62, and for *P. muralis,* 2.67 ± 0.75 (mean ± SD %) (Table 1).

No significant correlations between mean T_p_ and EWL_t_ were detected in either of the species, indicating a lack of support for a trade-off between these variables (*D. praticola*: R = −0.2; *p* = 0.580; *P. muralis*: R = 0.2; *p* = 0.580).

## 4. Discussion

Due to the limited data available for the *Darevskia* genus, we compared the thermal biology of the meadow lizard with that of other European lacertid species, focusing mainly on *P. muralis*. This species is the only small lacertid that shares a habitat with *D. praticola* at the western edge of its distribution range.

The results of this study suggest that *D. praticola* and *P. muralis* differ in some aspects of their ecophysiology. Regarding the thermal preference, we detected a significant difference, with *D. praticola* selecting lower temperatures than *P. muralis*. Its mean T_set_ in the laboratory (28.1 °C) is 2 °C lower than that of *P. muralis* (30.6 °C), from the present study and also lower than the mean set-point temperatures reported for 15 species of *Podarcis* lizards (ranging from 31.7 °C to 35.5 °C) [65]. Moreover, its set-point range (26.9–29.4 °C) falls mostly below that of *P. muralis* from the current study (29.0–32.7 °C), and completely below the values detected for the populations of the common wall lizard from the South of Europe (31.9–36.5 °C from Central Spain; 31.3–34.0 °C from Peloponnese, Greece; reported by Bauwens et al. [17] and Sagonas et al. [47], respectively). The mean T_p_ of *D. praticola* (28.1 °C) is more than 3 °C lower than that reported for *D. valentini* (31.5 °C) from Armenia [42]. On the other hand, in our previous research on the thermal ecology of *D. praticola,* we observed that its set-point temperature range closely resembles that of *Iberolacerta bonnali*, a cold-adapted alpine lizard. This similarity in T_set_ between a species living in temperate forests and an alpine species may be attributed to the relatively cool, shaded forest habitat of the meadow lizard, as well as the moderate temperatures during spring at this specific locality [41].

*Darevskia praticola* and *P. muralis* showed a different temporal pattern of daily fluctuations in T_p_. Probably due to their preference for higher temperatures, *P. muralis* showed a peak in T_p_ at the beginning of the experiment when they started thermoregulating. Later, they showed a stable pattern of body temperatures. In *D. praticola* we observed something peculiar. Until early afternoon, the lizards showed a stable pattern of body temperatures. However, after 15:00 their T_p_ started to decrease. This could be related to their daily activity pattern, as they are less active in the field in the afternoon. This pattern could also indicate a sensitivity to water loss. It could therefore be a compensatory mechanism for dehydration as the experiment progressed. This is consistent with the research of Sannolo et al. [15], where it was experimentally found that dehydrated lizards select lower temperatures in the gradient.

Our findings showed that the thermal preference of *D. praticola* was conserved within the same sex and season, as T_p_ and T_set_ were almost identical for males in the spring of 2014 [41] and 2018 (unpublished data) [66]. However, these temperatures also showed a potential for variation with seasonal weather, with lower values in the spring of 2019 (which was atypically cold and rainy); see Table 2. Additionally, the difference in T_set_ between the population of *P. muralis* from this study and those in Spain and Greece indicates some degree of plasticity of this trait. This is in accordance with previous research where it was shown that the thermal preference of a species can remain similar under different environmental conditions [3,19,20,21] but also shows plasticity for seasonal acclimatization [8,18,20,22].

Our main hypothesis was that *D. praticola* is sensitive to water loss, considering its preference towards forested and humid habitats, and that it has higher water loss rates than the widespread *P. muralis*. The patterns of water loss differed between the two investigated species regarding the time from the beginning of the experiment. In fact, *D. praticola* and *P. muralis* exhibited different trends in daily EWL patterns, even though the total EWL at the end of the experiment was almost the same. Various regulatory mechanisms can influence water loss. These may be structural, such as dynamic skin resistance to water loss [67], or behavioral, such as spending less time with eyes open to reduce water loss [68]. Water loss could also be increased by stress or increased physical activity, causing the animals to hyperventilate and lose more water through respiration. These mechanisms and induced physiological states, as well as possible intrinsic diurnal variations in activity and water loss, may lead to different temporal patterns of EWL during the experiment. *Darevskia praticola* showed a water loss pattern exhibiting an initially high EWL_i_, referred to as “initial acclimation”. This is a common EWL_i_ pattern observed in other lacertids, where the water loss is presumably higher due to the initial stress of handling the individuals [69]. As the experiment progressed, the EWL_i_ rate no longer fluctuated significantly and decreased towards the end of the experiment, probably due to reduced activity. *Podarcis muralis*, on the other hand, showed a pattern with a peak in the middle of the experiment, referred to as the “mid-peak” [69]. This pattern is still not well explained or understood, but a certain observation was made. In the work of Žagar et al. [69], this pattern was observed in diurnal lizards that live in mesic habitats and are active at ground surface. We hypothesize that the increased water loss in the early afternoon is a consequence of the daily activity cycle of this species. In spring, the activity of *P. muralis* is highest at this time because of the increased amount of sun exposure. So, they may experience an “internal clock” increase in activity and with it the increase in water loss during this time.

For the comparison of EWL rates, we used recent studies that applied the same experimental methodology, with a consideration of the different time frames of the measurements, which influence the total EWL. We compared the EWL values up to the eighth hour of the experiments to match the time frame of our experiment. EWL_t_ for *P. muralis* was 2.67%, which is lower than the EWL_t_ observed for *P. muralis* in Slovenia (3.12%—Osojnik et al. [24]) and higher than that reported for *P. muralis* in Spain (2.3%—Carneiro et al. [49]). These differences indicate the existence of local adaptations for this trait. Compared to other species, the EWL_t_ of both *D. praticola* and *P. muralis* was higher than those reported for some *Podarcis* and *Iberolacerta* species [24,25,49] but lower than those observed in some species of *Algyroides*, which are highly sensitive to water loss [5].

The low EWL_t_ value of *D. praticola* was peculiar, given the species’ preference for humid habitats and presumed sensibility to water loss. Comparing *D. praticola* temperature preference and evaporative water loss to other lacertid species showed that its preferred temperatures are lower than in most lacertids, while its EWL_t_ is moderate (but lower than expected). These results seem to indicate that it has developed mechanisms for water loss management and that its observed preference for humid habitats might be a result of its thermal constraints and the choice of more thermally favorable habitats. However, these findings may also be a result of the geographical origin of the species and its phylogeny, as it is the only representative of the *Darevskia* genus living in Europe. A large study conducted on the Lacertidae family showed that multiple lacertid clades have independently conquered cold environments [7]. These clades include the genus *Zootoca*, whose range extends into the Subarctic, as well as the montane genera *Iberolacerta*, *Darevskia,* and *Dinarolacerta*.

Our primary focus has been on the significance of ambient humidity and temperature for the activity patterns and the distribution of reptiles [4,5,6,7]. However, it is important to acknowledge that other environmental factors, such as vegetation cover, sun exposure, wind, and substrate humidity, also influence the thermal and hydric conditions of reptile microhabitats [70] and can directly impact their physiology [71,72]. Therefore, when assessing species’ thermoregulation and hydroregulation costs in the field, it is crucial to consider the variation of these environmental factors as well.

Given that *D. praticola* is a forest-dwelling lacertid lizard, we emphasize the importance of vegetation cover for its conservation. Forests mitigate the effects of the surrounding climate by creating distinct microclimatic conditions—characterized by lower maximum temperatures, higher minimum temperatures, and increased relative humidity compared to adjacent open habitats. This enables forest-dwelling species to be less reliant on broader climate patterns [73,74]. According to Maiorano et al. [75], most of the meadow lizard’s distribution range in Europe is situated in regions predicted to face a 70% to 90% risk of exposure to extreme climate conditions in the coming decades. Given this, ecophysiological studies such as this one could help in developing effective conservation strategies for the threatened species. For the meadow lizard, conservation efforts should focus on preserving the remaining natural habitats, particularly deciduous oak forests, which provide the necessary temperature and humidity conditions [53]. Additionally, in areas under intense anthropogenic alteration, conservation efforts should include the restoration of sufficiently large and interconnected suitable forest fragments.

## 5. Conclusions

Studies on the preferred temperatures showed that they tend to be more related to biogeographic origins and that the phylogenetic signal across Lacertidae indicates that T_p_ adaptations are characteristic of major clades that diverged early in lacertid evolution. It was also found that EWL was phylogenetically less conserved than T_p_, which may indicate a stronger selection pressure and faster adaptation of this physiological trait [7]. Thus, the “unusual” physiological traits of *D. praticola* may be a combination of historical influences, as well as recent local adaptations. This emphasizes the need for further studies on this species throughout its range as well as on other species of the genus.

## Figures and Tables

**Table 1:** Between-species comparison of main variables examined in this study. Sample size (N); mean, maximum, minimum, and standard deviation (SD) of the body mass (W_0_), snout-vent length (SVL), preferred body temperature (T_p_), and total accumulated evaporative water loss (EWL_t_) calculated for *Darevskia praticola* and *Podarcis muralis*. Mean ± standard deviation and range (in parenthesis). Note: The ranges of T_p_ are the min and max values from the whole data set, not of the T_p_ mean.

Species	N	W_0_ (g)	SVL (mm)	T_p_ (°C)	EWL_t_ (%)
*D. praticola*	10	2.35 ± 0.24 (1.97–2.64)	48.65 ± 1.70 (46.98–52.02)	28.1 ± 1.4 (21.0–32.8)	2.76 ± 0.62 (1.86–3.86)
*P. muralis*	10	4.95 ± 0.57 (3.91–5.79)	61.30 ± 2.85 (57.82–67.40)	30.6 ± 1.1 (21.9–36.7)	2.67 ± 0.75 (1.84–3.83)

**Table 2:** Preferred body temperatures (T_p_) and set-point temperatures (T_set_) for *Darevskia praticola* adult males, recorded in different years. Sample size (N).

Year	N	T_p_ (°C)	T_set_ (°C)
2014	19	22.1–35.4	27.8–31.4
2018	21	22.8–35.3	27.7–31.4
2019	10	21.0–32.8	26.9–29.4

## Data Availability

The raw data supporting the conclusions of this article will be made available by the authors upon request.

## Appendix A

This appendix contains three tables (Table A1, Table A2 and Table A3) with all values of T_p_, EWL_i,_ and EWL_a_ of *D. praticola* and *P. muralis* measured in these experiments.

**Table A1:** Daily variation of the preferred body temperatures (T_p_) of *Darevskia praticola* and *Podarcis muralis*.

SP	CODE	SVL	W0	TP9	TP10	TP11	TP12	TP13	TP14	TP15	TP16	TP17
DP	DP1	47.7	2.0	28.6	26.8	28.1	26.2	24.6	27.8	28.5	25.6	25.7
DP	DP2	49.7	2.6	29.2	28.7	28.5	26.0	29.8	23.5	27.8	26.9	22.6
DP	DP3	49.8	2.4	29.6	32.8	29.4	29.3	30.5	32.1	29.2	28.2	30.5
DP	DP4	47.2	2.3	28.2	31.2	26.6	32.6	29.2	29.2	28.9	28.8	30.9
DP	DP5	47.0	2.0	31.3	29.1	31.2	27.5	28.2	27.7	28.6	27.0	21.0
DP	DP6	48.2	2.6	29.2	31.0	29.9	28.5	26.2	29.1	28.7	26.8	23.3
DP	DP7	47.2	2.2	30.7	27.1	31.5	29.8	29.8	30.4	28.5	29.7	26.3
DP	DP8	47.5	2.4	23.7	29.8	28.6	23.2	31.1	29.3	30.1	23.1	27.3
DP	DP9	52.0	2.6	30.3	30.1	27.0	29.2	31.6	30.1	28.4	29.5	25.1
DP	DP10	50.2	2.4	25.9	27.6	23.5	27.7	27.1	28.2	25.6	24.5	21.1
PM	PM1	60.3	5.0	35.2	28.7	34.2	30.1	29.4	27.9	26.1	26.0	23.8
PM	PM2	57.8	4.0	30.8	29.7	34.1	24.5	31.8	34.3	31.7	34.6	29.1
PM	PM3	61.6	5.3	34.6	26.7	25.5	34.6	31.5	32.5	31.1	32.2	33.1
PM	PM4	59.8	5.2	35.5	32.3	22.1	35.7	32.5	32.1	33.7	30.2	31.2
PM	PM5	64.1	5.2	34.1	21.9	25.4	28.6	30.7	27.6	36.7	28.7	28.3
PM	PM6	60.5	5.1	33.6	24.7	35.3	30.2	27.2	33.1	33.3	29.5	32.0
PM	PM7	62.2	5.1	32.8	29.9	30.4	29.8	30.8	22.6	34.6	22.6	33.8
PM	PM8	61.3	5.1	32.7	33.6	30.9	22.4	35.1	22.4	26.1	30.4	32.7
PM	PM9	58.0	3.9	35.2	32.3	35.8	30.8	32.1	32.0	24.6	28.1	28.2
PM	PM10	67.4	5.8	30.2	33.6	36.1	36.2	30.3	32.5	34.2	33.2	24.7

SP—species: DP—*Darevskia praticola*, PM—*Podarcis muralis*; SVL—snout to vent length (mm); W0—initial weight (g); TP9–TP17—preferred body temperatures (°C) from 09:00 to 17:00 h.

**Table A2:** Daily variation of the instantaneous water loss (EWL_i_) of *Darevskia praticola* and *Podarcis muralis*.

SP	CODE	SVL	W0	EWLi 9–10	EWLi 10–11	EWLi 11–12	EWLi 12–13	EWLi 13–14	EWLi 14–15	EWLi 15–16	EWLi 16–17
DP	DP1	47.7	2.0	0.41	0.51	0.20	0.20	0.10	0.51	0.46	0.05
DP	DP2	49.7	2.6	0.55	0.19	0.16	0.35	0.51	0.35	0.19	0.12
DP	DP3	49.8	2.4	0.58	0.41	0.33	0.37	0.08	0.41	0.41	0.25
DP	DP4	47.2	2.3	0.47	0.34	0.34	0.21	0.17	0.26	0.26	0.30
DP	DP5	47.0	2.0	0.46	0.61	0.71	0.25	0.10	0.76	0.51	0.46
DP	DP6	48.2	2.6	0.62	0.27	0.27	0.12	0.16	0.39	0.23	0.12
DP	DP7	47.2	2.2	0.73	0.14	0.60	0.60	0.32	0.69	0.23	0.09
DP	DP8	47.5	2.4	0.86	0.33	0.16	0.41	0.29	0.70	0.20	0.20
DP	DP9	52.0	2.6	0.27	0.30	0.08	0.38	0.42	0.15	0.23	0.08
DP	DP10	50.2	2.4	0.86	0.41	0.08	0.41	0.33	0.45	0.41	0.25
PM	PM1	60.3	5.0	0.60	0.26	0.04	0.20	0.40	0.10	0.36	0.06
PM	PM2	57.8	4.0	0.43	0.55	0.20	0.45	0.48	0.27	0.40	0.30
PM	PM3	61.6	5.3	0.32	0.40	0.38	0.38	0.09	0.30	0.27	0.30
PM	PM4	59.8	5.2	0.27	0.41	0.21	0.99	0.58	0.50	0.47	0.31
PM	PM5	64.1	5.2	0.23	0.33	0.25	0.33	0.25	0.23	0.06	0.15
PM	PM6	60.5	5.1	0.39	0.43	0.28	1.10	0.51	0.57	0.43	0.10
PM	PM7	62.2	5.1	0.51	0.35	0.06	0.55	1.14	0.27	0.22	0.18
PM	PM8	61.3	5.1	0.59	0.41	0.06	0.33	0.63	0.04	0.12	0.33
PM	PM9	58.0	3.9	0.56	0.38	0.08	0.15	0.72	0.05	0.10	0.05
PM	PM10	67.4	5.8	0.33	0.21	0.16	0.45	0.38	0.03	0.12	0.21

SP—species: DP—*Darevskia praticola*, PM—*Podarcis muralis*; SVL—snout to vent length (mm); W0—initial weight (g); EWLi—instantaneous water loss between time intervals from 09:00 to 17:00 h.

**Table A3:** Daily variation of the accumulated water loss (EWL_a_) of *Darevskia praticola* and *Podarcis muralis*.

SP	CODE	SVL	W0	EWLa 10	EWLa 11	EWLa 12	EWLa 13	EWLa 14	EWLa 15	EWLa 16	EWLa 17
DP	DP1	47.7	2.0	0.4	0.9	1.1	1.3	1.4	1.9	2.4	2.4
DP	DP2	49.7	2.6	0.5	0.7	0.9	1.2	1.8	2.1	2.3	2.4
DP	DP3	49.8	2.4	0.6	1.0	1.3	1.7	1.8	2.1	2.6	2.8
DP	DP4	47.2	2.3	0.5	0.8	1.2	1.4	1.5	1.8	2.1	2.4
DP	DP5	47.0	2.0	0.5	1.1	1.8	2.0	2.1	2.9	3.4	3.9
DP	DP6	48.2	2.6	0.6	0.9	1.2	1.3	1.4	1.8	2.1	2.2
DP	DP7	47.2	2.2	0.7	0.9	1.5	2.1	2.4	3.1	3.3	3.4
DP	DP8	47.5	2.4	0.9	1.2	1.4	1.8	2.0	2.7	2.9	3.2
DP	DP9	52.0	2.6	0.3	0.6	0.6	1.0	1.4	1.6	1.8	1.9
DP	DP10	50.2	2.4	0.9	1.3	1.4	1.8	2.1	2.5	2.9	3.2
PM	PM1	60.3	5.0	0.6	0.9	0.9	1.1	1.5	1.6	2.0	2.0
PM	PM2	57.8	4.0	0.4	1.0	1.2	1.6	2.1	2.4	2.8	3.1
PM	PM3	61.6	5.3	0.3	0.7	1.1	1.5	1.6	1.9	2.1	2.4
PM	PM4	59.8	5.2	0.3	0.7	0.9	1.9	2.5	3.0	3.4	3.7
PM	PM5	64.1	5.2	0.2	0.6	0.8	1.1	1.4	1.6	1.7	1.8
PM	PM6	60.5	5.1	0.4	0.8	1.1	2.2	2.7	3.3	3.7	3.8
PM	PM7	62.2	5.1	0.5	0.9	0.9	1.5	2.6	2.9	3.1	3.3
PM	PM8	61.3	5.1	0.6	1.0	1.1	1.4	2.0	2.1	2.2	2.5
PM	PM9	58.0	3.9	0.6	0.9	1.0	1.2	1.9	1.9	2.0	2.1
PM	PM10	67.4	5.8	0.3	0.5	0.7	1.1	1.5	1.6	1.7	1.9

SP—species: DP—*Darevskia praticola*, PM—*Podarcis muralis*; SVL—snout to vent length (mm); W0—initial weight (g); EWLa—accumulated water loss from 10:00 to 17:00 h.
